# Digital health technologies need regulation and reimbursement that enable flexible interactions and groupings

**DOI:** 10.1038/s41746-024-01147-z

**Published:** 2024-06-18

**Authors:** Rebecca Mathias, Peter McCulloch, Anastasia Chalkidou, Stephen Gilbert

**Affiliations:** 1 Else Kröner Fresenius Center for Digital Health, TUD Dresden University of Technology, Dresden, Germany; 2 Nuffield Department of Surgical Sciences, University of Oxford, Oxford, UK; 3 National Institute for Health and Care Excellence (NICE), London, UK

**Keywords:** Health policy, Health care economics

## Abstract

Digital Health Technologies (DHTs) are being applied in a widening range of scenarios in medicine. We describe the emerging phenomenon of the grouping of individual DHTs, with a clinical use case and regulatory approval in their own right, into packages to perform specific clinical tasks in defined settings. Example groupings include suites of devices for remote monitoring, or for smart clinics. In this first article of a two-article series, we describe challenges in implementation and limitations in frameworks for the regulation, health technology assessment, and reimbursement of these device suites and linked novel care pathways.

Digital health technologies (DHTs) include wearables and devices for mobile health (mHealth), health information technology (IT), telehealth and telemedicine, and personalized medicine^1,2^. There is a global trend for the greater utilization of these technologies in medical practice. However, there are substantial national differences in how they are perceived, evaluated for regulatory approval, and reimbursed or otherwise compensated^3^. DHTs will play an increasing role in the economic affordability and environmental sustainability of healthcare systems with the capacity to meet societal needs that were previously unaddressed^4,5^. By offering customized, adaptable care solutions, they hold the potential to facilitate safer and more extensive home-based and local care while empowering patients to actively engage in their health management^6^. In this first article in a two-article series, we describe the new phenomenon of flexible suites of DHTs, the currently applicable regulatory and HTA frameworks, and their application during the COVID-19 pandemic. In the second article of the series, we describe what new frameworks could include.

Since the COVID-19 pandemic, there has been increasing bundling of individual DHTs, which have specified intended medical purposes and associated approvals, into suites to deliver aggregated ‘intended purposes’ in some cases different from those of any individual component device. The complexity of the interactions between devices within suites and of suites with one another, can deliver new paradigms of medical treatment but may also produce unexpected “emergent” actions or properties. Sets of DHTs can have differing levels of workflow integration with telemedicine and with the electronic health record (EHR). Examples of services delivered through device-aggregates of DHTs are groupings of remote passive and active sensor systems, automated monitoring platforms, alarms, and teleconsultation platforms to (i) allow Health Care Providers (HCP) to monitor chronically ill patients at home^4^; (ii) enable Hospital-at-Home programs (HaH)^7^ (iii) improve treatment efficiency in hospital or care home settings^8,9^; and, (iv) enable ‘smart clinics’ with physical exams by remote clinicians via the patient or their family in their own home by the use of mHealth platforms and wireless thermometers, stethoscopes, otoscopes and laryngoscopes^10^ (Fig. 1). We examine the challenges posed by the implementation, evaluation, and assessment of interactions within grouped systems of networked devices for regulatory and health technology assessment (HTA) purposes.

**Fig. 1:**
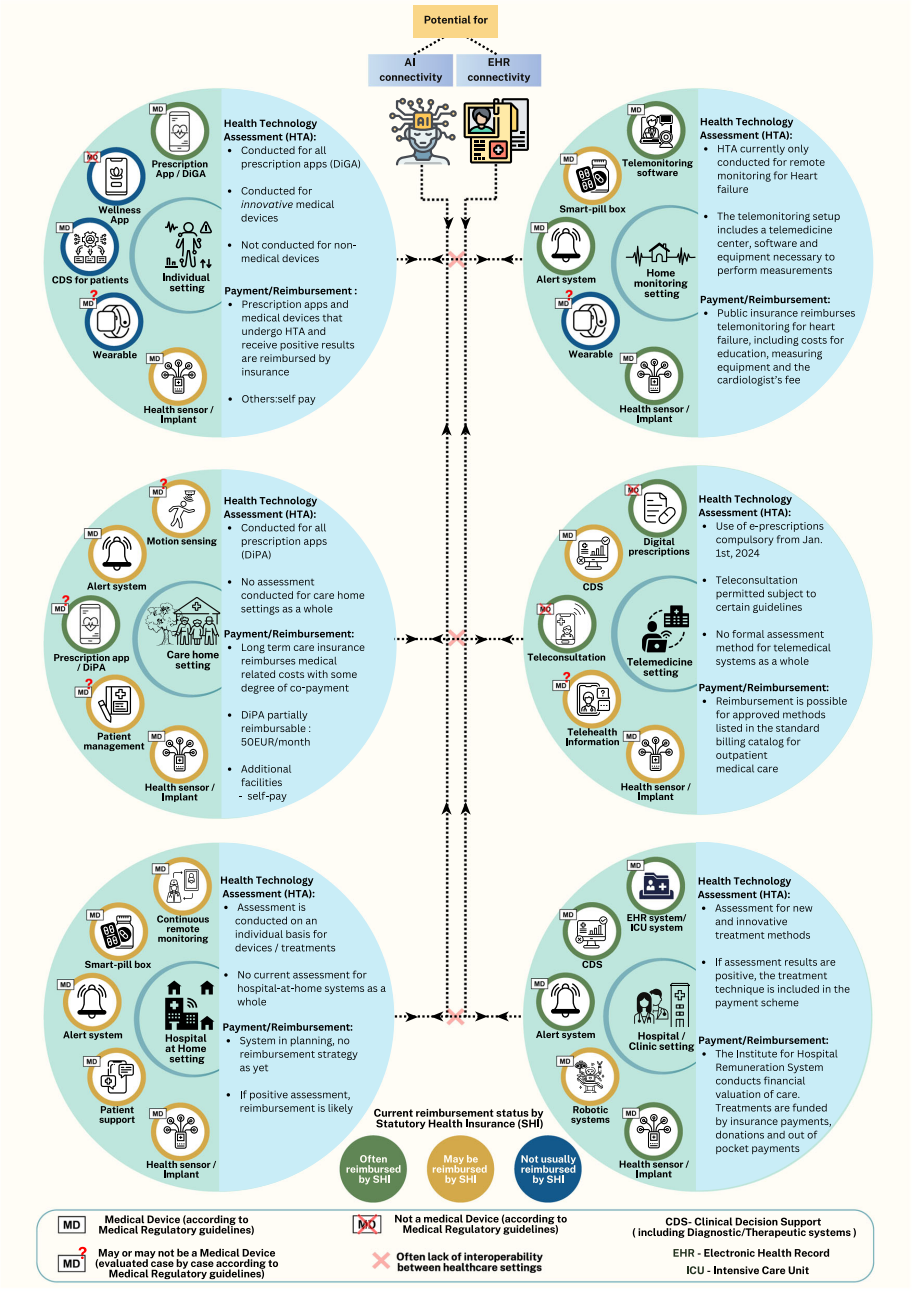
Overview of modular DHT use across different healthcare settings. Examples of emerging approaches in the international adoption of suites of DHT devices are shown, along with their component devices and applicable regulatory and HTA frameworks^26^. The frameworks are described for Germany, which has a statutory health insurance system, and a novel reimbursement strategy for digital applications^27^. Each circle represents a different healthcare setting, with icons inside representing various DHTs that could be grouped to interact within that setting. The colors around the icons signify reimbursement status in Germany: green for often reimbursed, yellow for possible reimbursement, and blue for not usually reimbursed. The dotted lines illustrate potential connectivity between devices, healthcare settings, electronic health records, and AI tools. The red crosses indicate a lack of interoperability between these systems, highlighting the need for improved connectivity and interoperability for comprehensive patient health records and treatment decisions.

## Have grouped devices not always existed?

On one level, the ad hoc grouping together of separate medical devices which were developed by different manufactures is a long-standing practice. For example, an orthopedic surgeon’s preferred instruments and/or implants are often grouped and sterilized together in racks or trays. In some cases, such groupings were brought together, and regulated together as ‘procedure packs’^11^. A key difference between ‘sets’ of such ‘traditional’ medical devices and aggregations of DHTs, is that the latter are used to build a suite of networked interconnected devices, which have complex data flows and resultant dynamic dependencies between each other. While many suites continue to be reliant on healthcare staff for operation, there is realistic potential for transitioning to greater automation and use of these as aggregated ‘super devices’ with limited human intervention in the future.

## The HaH concept as an example of the evolution of DHT/HCP aggregate systems

As described in Fig. 1, the Hospital at Home (HaH) suite generally consists of aggregated health sensors, alert systems, smart pill boxes, systems for continuous remote monitoring, and remote digital patient support. This application is already used to treat patients, so by definition, there already are processes in place for its regulation. Although the HaH concept existed before the COVID-19 pandemic, it was during the pandemic that the concept gained traction in the UK and several other countries^12^. This was due, at least in part, to temporary regulation, and reimbursement changes globally. In the US, the Centers for Medicare and Medicaid Services (CMS), allowed for the reimbursement of HAH care as a diagnosis-related group (DRG) as well as waived certain previously mandatory requirements for remote patient monitoring^13^. Other regulatory changes included encouraging the adoption of telehealth and remote consulting, allowing for remote acquisition of data, along with fast-tracking of trials and of the entry of vaccines into the market^14,15^. Countries that have applied the HaH model are the UK, US, Spain, The Netherlands, Canada, and Australia, and there has also been early adoption in many other european countries^7^. In the US, the CMS has extended the acute HaH program, initiated in 2019 during the pandemic, until Dec 2024 to further evaluate the benefits of HAH, marking a pioneering step towards telehealth adoption^16^. A 2023 early value assessment by the National Institute for Health and Care Excellence (NICE), in the UK, supported the potential cost-effectiveness of HAH or virtual wards, while acknowledging the remaining uncertainty and need for further evidence generation. That review also highlighted assessment challenges such as insufficient prior data, a lack of suitable comparators, interactions across diverse policies, variations in the standard of care, and overall heterogeneity^17,18^. However, suited to the rapid pace of digital innovation, NICE’s conditional approval supported both the ability to safely generate further evidence and the addressing of unmet patient needs.

## Challenges for adoption and evaluation of DHT suites

There are safety and performance challenges with the grouping of DHTs, especially those built up of individual medical device (MD) and non-MD DHTs, due to networked-device interactions, and other emergent system properties^19^. Ensuring interoperability and applicability across various demographics poses significant hurdles, necessitating a thorough examination of each system component. Cybersecurity of individual components and of the aggregated system of DHTs, including internet of Medical Things devices (IoMT, e.g., sensors), requires careful regulatory attention, particularly for systems that will form part of clinical health delivery infrastructure, such as HaH services, as the broader risks they pose could extend beyond individual patients and threaten the integrity of entire healthcare systems^20^. Many DHTs are adaptive, either through agile software refinements, or the ongoing training of artificial intelligence models, and this brings additional complexity^21^.

In the EU, medical device regulations require that systems of medical devices (MD) and non-MD DHTs provided by commercial providers be treated as MDs by regulatory authorities^22^ (Article 22). The commercial provider must then specifically risk assess all components, with the provision of clinical data for the combination^23^. Based on this, the interpretation of EU law could suggest that manufacturers incorporating built-in smartphone/smartwatch components like ECG sensors might require MD approval for all components, even those they don’t produce themselves, which could be impractical given their non-manufacturer status. Guidance attempts clarification but burdens app developers with assessing and providing evidence for every sensor configuration, a daunting task given the multitude of smartphone brands and sensors^23^. However, as the flexibility and variability of clinical settings in which devices may be grouped into suites increases, it is valid to ask the question, of whether the only model that could deliver benefit and safety for patients is requiring developers to anticipate and test for every possible scenario of interaction. Reimbursement of such systems also presents a challenge. Assessing cost-effectiveness and gathering evidence for extensive modular DHT suites introduces new complexities. These include the absence of comparators and the complex estimation of initial investments required for staff training and system development^18^.

## Summary

Much of the transformation that will come from DHTs in increasing the quality and efficiency of care will come from novel suites of DHTs in interactive ‘fuzzy’ care networks with HCPs, and not from individual DHTs. In the US, the Center for Devices and Radiological Health (CDRH) is actively working on forward-thinking strategies for a regulatory pathway that integrates this line of thought^24^. There will be further maturation of the concept of suites of DHTs, and further economic evaluations of these concepts. Although temporary adaptations of regulatory and HTA frameworks served well in the pandemic, there is an opportunity to explore whether new frameworks and even new paradigms can be developed for the novel scenario of complex multi-device digital/human workflows. We will address the question of what new frameworks could be included in the second article of this two-article series^25^.
